# Resiliency of healthcare expenditure to income shock: Evidence from dynamic heterogeneous panels

**DOI:** 10.3389/fpubh.2023.1085338

**Published:** 2023-03-07

**Authors:** Shafiun Nahin Shimul, Muhammad Ihsan- Ul- Kabir, Fariha Kadir

**Affiliations:** Institute of Health Economics, University of Dhaka, Dhaka, Bangladesh

**Keywords:** healthcare expenditure, GDP, income elasticity, Dynamic Fixed Effects, mean group, pooled mean group, income shock

## Abstract

Using the World Bank data over the period of 1960–2019, this study aims at estimating the resiliency of health expenditures against gross domestic product (GDP). Long-run and short-run elasticities are calculated using the type of panel time series methods that are exclusively designed for dynamic heterogeneous panels: Mean Group, Pooled Mean Group, and Dynamic Fixed Effects estimators. These methods permit better estimations of elasticity with considerable heterogeneity across the 177 countries included in this study. Along with a standard elasticity estimation, this study estimates country-specific long-run and short-run elasticities along with error correction components. The study finds that the long-run elasticity of income is very close to unity, but short-run coefficients are insignificant for most nations. In addition, most countries revert to long-run equilibrium reasonably quickly if there is shock as the error correction coefficients are negative and, in many cases, very close to one. While for most developed countries, the short-run elasticities are lower in comparison with the short-run elasticities of developing countries indicating that many developing countries may face a larger decrease in health expenditure with the forecasted decline in income due to impending economic recession. Therefore, although this study is not directly intended to capture the post-COVID-19 effects, the study estimates may project the potential responses in health expenditure across countries due to potential income shocks.

## 1. Introduction

This paper aims at estimating the income elasticity of healthcare expenditure using data from the past 60 years for 177 countries across the world to recognize how healthcare expenditures respond to economic fluctuations. With this estimate, we can gain insight into the stability and resilience properties of healthcare spending. For instance, if healthcare expenditures are elastic (i.e., healthcare as a luxury good) with respect to changes in GDP, then with economic downfall, healthcare spending will fall more than proportionately, destabilizing the countries' healthcare spending.

Conversely, if healthcare expenditures are inelastic (i.e., healthcare is a necessary good), it would indicate that health expenditure will not fluctuate significantly with changes in income. With this estimate, we can assess the resiliency of healthcare expenditures both in absorbing an instantaneous shock from an economic impact and in estimating the time needed to revert back to its long-run equilibrium once deviates from the equilibrium. Estimation of such relationships is widely available; however, most of the previous studies attempted to understand this relationship using fixed effects ignoring an essential nature of heterogeneity across panels. Most importantly, many earlier studies fail to provide common long-run coefficients for all countries with heterogeneous short-term coefficients as they tend to assume homogeneity. Using the Pool Mean Group (PMG) estimation method—a suitable estimation method for common long-run estimates as well as heterogeneous short-run estimates—this study provides both aggregate and country-specific long-term and short-term estimates.

In doing so, we use panel data of current health expenditure (CHE) per capita, GDP per capita, out-of-pocket (OOP) expenditure (% of CHE), and life expectancy (LE) at birth for 177 countries from 1960 to 2019. We rely mostly on PMG estimates as this estimation method provides aggregate level long-run efficiency, which is believed to be relatively stable, with country-specific fluctuating short-run coefficients. We use Mean Group (MG) and PMG estimators designed explicitly for estimating long-run relationships from dynamic heterogeneous panels. Although PMG is exclusively designed for estimation in the case of a heterogeneous panel, based on our knowledge, these tools are rarely applied to explore the relationship between GDP and healthcare expenditure. However, we also use the more conventional Dynamic Fixed Effects (DFE) estimator for comparison. Since with these estimates, we can understand the common overall long-term trajectory of health expenditure along with short-term fluctuations and resiliency, these results can provide insights into the potential outcomes of an income shock. Therefore, while this study does not directly address COVID-19 issues, key findings of this study will leave strong implications on what to expect with regard to fluctuations in healthcare expenditure across countries under COVID-19-related GDP shocks. Hence, the current study will have strong policy implications on health expenditure and its relationship to income shocks.

The paper is organized as follows. Section 2 covers related literature; Section 3 includes data and estimation methods. Section 4 provides findings and discussions, and Section 5 provides conclusions and policy recommendations.

## 2. Related literature

The income elasticity of healthcare expenditure, as evident in the literature, often surpassed unity, indicating healthcare as a luxury good (1–7). Much of these works have been grounded on cross-country data and recently with panel data followed by unit root tests and cointegration analysis carried out, especially for developed countries. The most notable work in this issue is that of Newhouse that used 1 year of cross-sectional data from 13 developed countries and estimated an elasticity exceeding one (2). Newhouse observed that over 90% of the variation between countries in per capita healthcare expenditure could be explained by variations in per capita GDP, with an income elasticity ranging from 1.15 to 1.31. Later, Newhouse promotes that there is a substantial role for organizational factors of healthcare delivery and financing in determining healthcare expenditures (8).

Parkin et al. (9) show that different conversion factors (exchange rates and healthcare purchasing power parities, PPP) lead to different results with respect to the estimated income elasticity of healthcare expenditure, and the use of healthcare PPP reduces the income elasticity below unity (0.9). In contrast, Gerdtham et al. (10) suggest that the value of estimated income elasticity is invariant with respect to the use of GDP or healthcare spending, although the use of exchange rate adjustment leads to a trivial fall in estimated elasticity. Hitiris and Posnett (11) re-examine the results of previous work covering 20 OECD countries and find GDP as a determinant of healthcare expenditure, with an estimated income elasticity at or around unity, and propose that OECD countries should not be regarded as a single, homogeneous group. Though this study acknowledges heterogeneity, no attempt has been made to estimate the parameters considering heterogeneity.

Moore et al. (12) find that income is the most dominant determinant of healthcare spending, which explains above 90% of the variance in expenditures across 20 OECD countries, and observed that long-run income elasticity of medical care exceeds unity, in accordance with Culyer (13). Using panel data, other studies find elastic healthcare spending (14–16).

Using country-specific time series data, multiple studies find that the income elasticity of healthcare spending is greater than unity (17–20). Blomqvist and Carter (21) claim that when comprehensive data are used, health expenditure cannot be considered a luxury product. Getzen (22) posits that the debate arises primarily from mis-specifying the levels of analysis—between vs. within estimates. The study finds that individual income elasticities are usually near zero with social security, while national healthcare expenditure elasticities are usually greater than unity. Hence, he summarizes that “healthcare is an individual necessity and a national luxury”. Another group of researchers, Clemente et al. (23), show a long-term relationship between the total healthcare expenditure and gross domestic product (GDP) using the cointegration approach and state that potential non-stationarity of data and cross-section heterogeneity may serve as the reasons behind healthcare expenditure being more than unity. Correspondingly, Jewell et al. (24) indicate that before studying the relationship between healthcare expenditure and income, it is critical to specify whether these variables are stationary. In empirical tests, disregarding the above issues will lead to pointless results and spurious regression (25, 26).

In different circumstances, a number of studies of income elasticity for healthcare spending produce estimates of less than unity (27–30). Matteo (28) provides a comparison between parametric and non-parametric estimation techniques. He shows that locally weighted scatterplot smoothing allows for variability in the income elasticity of health albeit inapt for multivariate cases. However, this limitation can be partially addressed by combining non-parametric estimators with parametric specifications (31).

Later, Panel Smooth Threshold Regression has been developed to indicate a change in parameters among countries and also change in parameters over time (32–35). Using this approach, Mehrara et al. (36) estimate the relationship between healthcare expenditure and income for 16 OECD countries and reveal that income elasticity is much more than unity (2.59) and also the estimation has been unvarying over time and across countries.

Convergence of healthcare expenditure by applying economic growth models in developed countries has been examined in some previous studies (37–44). However, Barros (38) finds that the characteristics of health systems (e.g., availability of gatekeepers, public reimbursement) have no significant effects on either the growth or level of health expenditure. Nghiem and Connelly (45) also reveals no evidence that the growth of health spending per capita in OECD countries converges over time.

While the income elasticity of healthcare spending remains inconclusive for developed countries, it is rarely explored for less developed countries. By using panel data, some studies of developing countries indicate that healthcare is a necessity rather than a luxury, and healthcare expenditure in general does not grow faster than GDP after taking other factors into consideration (46–50). Furthermore, Farag et al. (47), find that healthcare spending is least responsive to changes in income in low-income countries and most responsive in middle-income countries, in comparison with high-income countries falling in the midway.

In a recent study, Stepovic (51) confirms that there had always been differences between low and high-income countries in the speed of recovery. Abdullah et al. (52) conduct a study of 36 Asian countries and find that long-run income elasticity of healthcare expenditure is less than unity. The findings collide with Hassan et al. (53) but are in line with some other studies (36, 54–56). In another study using the panel data method; Baltagi et al. (57) stated that the size of income elasticity depends on the geo-political position of different countries in the global income distribution, with poorer countries showing higher elasticity. Obradović and Lojanica (58) accomplish a study on South-Eastern European Health Network countries which shows that in the long run, the income elasticity of healthcare expenditure is greater than unity and states healthcare can be considered a luxury good. Additionally, the study reveals that the elasticity of healthcare expenditure relative to income is less than unity in the short run, which means that healthcare is a necessary product over the short term.

Using dynamic panel data, a reciprocal relationship has been found between health expenditure and economic growth in the short run and one-way causality from economic growth to public health expenditure in the long run (59). Rana et al. (60) examine the common correlated effects on income elasticity and health expenditure using the mean group (MG) method. Findings show that about 43% of the variation in global health expenditure growth can be explained by economic growth. Income shocks affect the health expenditure of high-income countries more than lower-income countries. Moreover, the income elasticity of health expenditure is less than one for all income levels. Similar to prior studies, Murthy and Okunade (61) present empirical evidence that in the U.S. health care is a necessity, along with an income elasticity estimate of around 0.92.

To elucidate the context of Asian countries, Mehmood et al. (62) estimate the presence of a long run relationship between income per capita, health expenditures, and health literacy using pooled mean group (PMG) estimation method for a sample of 26 Asian countries (1990–2012). Alhassan et al. (63) uses Pesaran's autoregressive distributed lag model on annual time-series data from Nigeria to conceptualize the hypothesized claim about the sustaining relationship between economic growth and public health expenditure. The empirical findings experience the long-run relationship between public health expenditure and economic growth over the entire study span.

Iheoma (64) employs the panel autoregressive distributed lag model to express the theoretical relationship between public health expenditure per capita, economic uncertainty, and population growth rate. Using the mean group (MG) and the pooled mean group (PMG) estimators, the study reveals that in low-income countries, economic uncertainty is negatively associated with health spending in the short run. In lower-middle-income countries, economic uncertainty increases health spending in the short run but reduces it in the long run as uncertainty persists. Fedeli (65) upholds the view that an increase in GDP accelerates healthcare expenditure in both the long and the short run, although at a decreasing rate in the short run.

The common drawbacks of most of the previous studies are reliance on relatively small-size homogenous samples and using relatively weak or less suitable econometric modeling with available data sets. Moreover, the majority of the previous studies either estimated a single long-run and short-run estimates, or separate estimates for each country. None of them utilizes the strength of the PMG approach which capitalizes on the strength of panel regression by proving a common long-run coefficient with varying short-term coefficients across countries. The current study overcomes those limitations.

A major contribution of the present paper is that it applies panel estimation methods for healthcare expenditure and GDP taking heterogeneity among countries into consideration, thus providing rigorous and robust elasticity^1^ estimates of healthcare spending and analyzing observed heterogeneity across countries' healthcare expenditure systems.

## 3. Data and estimation method

In our empirical estimations, we use values of CHE per capita, GDP per capita, OOP (% of CHE), and life expectancy at birth taken from the World Development Indicators. Healthcare expenditure and GDP data cover the period 1960–2019 for 177 countries. CHE per capita is measured in current US dollars and includes healthcare goods and services consumed during each year. GDP per capita is gross domestic product divided by midyear population and data are in current U.S. dollars. OOP (% of CHE) is the share of out-of-pocket payments of total current health expenditures whereas out-of-pocket payments are spending on health directly out-of-pocket by households. Life expectancy at birth indicates the number of years a newborn infant would live if prevailing patterns of mortality at the time of its birth were to stay the same throughout its life.

To leverage the strength of panel data, we use MG and PMG dynamic panel estimators which are applied to account for heterogeneity among countries in panel data sets. For reference, we also apply DFE estimation methods.

There has been growing interest in dynamic panel data models, where the number of time series observations, *T*, is relatively large and of the same order of magnitude as *N*, the number of groups. Pesaran et al. (66) report that the usual practice is either to estimate *N* separate regressions and then compute the mean of estimated coefficients, which demonstrate an MG estimator, or to pool data assuming that slope coefficients and error variances are identical, as with the DFE method. They indicate an intermediate procedure, the PMG estimator, which constrains long-run coefficients to be identical but allows short-run coefficients and error variances to vary across groups. Both cases are considerable when regressors are non-stationary and they follow unit root processes, and for both cases derive the asymptotic distribution of PMG estimators as *T* tends to infinity.

Subsequently, we employ a traditional DFE estimator along with dynamic panel MG and PMG estimation. DFE estimates the time series for each group pooled and only intercepts are allowed to vary across groups. However, there are no grounds to assume that the rate of convergence to the steady state is identical across countries, as the DFE method assumes. MG estimator relies on estimating *N* time series regressions and averaging the coefficients (25). This method generates consistent estimates of parameter averages, yet it does not allow for the possibility that certain parameters may be analogous across groups. In contrast, the PMG estimator is an intermediate estimator since it uses a combination of pooling and averaging of the coefficients. A PMG estimator allows the intercepts, short-run coefficients, and error variances to differ across groups (as would an MG estimator) but constrains the long-run coefficients to be equal (as would a DFE estimator). MG estimators provide consistent estimates of the mean of long-run coefficients, though these will be inefficient if slope homogeneity holds. Under long-run slope homogeneity, the pooled estimators are consistent and efficient. Even so, the long-run slope homogeneity imposed by PMG can be easily tested using the Hausman test (67). There is no reason to believe that in such a large panel there would not be substantial heterogeneity, and therefore, any estimation tool that takes this issue into consideration should be used for estimation. Both MG and PMG estimators are intended to deal with panel data characterized by a large number of groups *N* and a large number of time periods *T*, as the data used in this paper. However, PMG estimators appear to be more relevant in our case because it is very likely many countries will follow a similar long-run trend keeping the avenue open for heterogeneity in short-run estimates.

Given data in time periods, *t* = 1, 2, …, *T*, and groups, *i* = 1, 2, …, *N*, Pesaran et al. (66) estimated an *ARDL (p, q, q, …, q)* model,


(1) $$\begin{array}{l} y_{it}=\sum_{j=1}^{p}\lambda_{ij}y_{i,t-j}+\;\sum_{j=0}^{q}\delta_{ij}^{\prime }X_{it}+\mu_{i}+\epsilon_{it} \end{array}$$

where **X**_*it*_ (*k* × 1) is the vector of explanatory variables (regressors) for group *i*; μ_*i*_ represents the fixed effects; the coefficients of lagged dependent variables, λ_*ij*_, are scalars; $\delta_{ij}^{\prime }$ are (*k* × 1) coefficient vectors; and *T* must be large enough such that the model can be estimated for each group separately. Similarly, time trends and other types of fixed regressors can be included in Equation (1).

If variables in Equation (1) are, for example, *I*(1) and cointegrated, then the error term is an *I*(0) process for all *i*. A prime feature of cointegrated variables is their responsiveness to any aberration from long-run equilibrium. This feature entails an error correction model in which the short-run dynamics of variables in the system are influenced by aberration from equilibrium. Thus, it is convenient to work with the reparameterization of Equation (1) into the error correction equation,


(2) $$\begin{array}{l} \Delta y_{it}=\phi_{i}y_{i,t-1}+\;{\beta^{\prime }}_{i}X_{it}+\sum_{j=1}^{p-1}\lambda_{ij}^{*}\Delta y_{i,t-j}+\sum_{j=0}^{q-1}\delta_{ij}^{{*}^{\prime }}\Delta X_{i,t-j}\\ +\;\mu_{i}+\epsilon_{it}\\ i=1,2,\ldots .,N\;,\;\text{and }t=1,2,\ldots ,T,\;\text{where }\\ \phi_{i}=-\left(1-\;\sum_{j=1}^{p}\lambda_{ij}\right),\beta_{i}=\;\sum_{j=\;0}^{q}\delta_{ij},\\ \lambda_{ij}^{*}=-\sum_{m=j+1}^{p}\lambda_{im},j=1,2,\ldots ,p-1,\;\text{and }\\ \delta_{ij}^{*}=\;-\sum_{m=j+1}^{q}\delta_{im},\;j=1,2,\ldots ,q-1. \end{array}$$

The parameter ϕ_*i*_ is the error-correcting speed of the adjustment term. If ϕ_*i*_ = 0, there would be no evidence for a long-run relationship. This parameter is expected to be significantly negative under the prior assumption that the variables return to long-run equilibrium. Notably, the vector ${\beta^{\prime }}_{i}$ comprises long-run relationships between the variables.

If time series observations are considered for each group, Equation (2) can be written as:


(3) $$\begin{array}{l} \Delta y_{i}=\phi_{i}y_{i,-1}+X_{i}\beta_{i}+\;\sum_{j=1}^{p-1}\lambda_{ij}^{*}\Delta y_{i,-j}+\sum_{j=0}^{q-1}\Delta X_{i,-j}\delta_{ij}^{*}\\ +\;\mu_{il}l+\epsilon_{i} \end{array}$$

where, $i=1,2,\ldots ,N;\;y_{i}={\left(y_{i1},\;y_{i2},\ldots ,y_{iT}\right)}^{\prime }$ is a *T*×*1* vector of observations on the dependent variable of *i*-th group; $X_{i}={\left(x_{i1},x_{i2},\ldots ,x_{iT}\right)}^{\prime }$ a *T*×*K* matrix of observations on regressors that vary both across groups and time periods; *l* = (1, 1, …, 1)′ a *T*×*1* vector of ones; *y*_*i*, −*j*_ and *X*_*i*, −*j*_ are *j* period lagged values of *y*_*i*_ and *X*_*i*_; Δ*y*_*i*_ = *y*_*i*_−*y*_*i*−1_; Δ*X*_*i*_ = *X*_*i*_−*X*_*i*−1_; Δ*y*_*i*, −*j*_ and Δ*X*_*i*, −*j*_ are *j* period lagged values of *y*_*i*_ and *X*_*i*_; and $\epsilon_{i}=\;{\left(\epsilon_{i1},\epsilon_{i2,}\ldots ,\epsilon_{iT}\right)}^{\prime }$.

To estimate the model, Pesaran et al. (66) adopted a likelihood approach assuming that the disturbances ϵ_*it*_ are normally distributed. The parameters of interest are long-run effects and adjustment coefficients. Expressing the likelihood of panel data as the product of likelihoods for each group and taking the log yields:


$$\begin{array}{l} l_{T}\left(\varphi \right)=-\frac{T}{2}\sum_{i=1}^{N}ln2\pi \sigma_{i}^{2}\\ -\frac{1}{2}\;\sum_{i=1}^{N}\frac{1}{\sigma_{i}^{2}}{\left(\Delta y_{i}-\phi_{i}\xi_{i}\left(\theta \right)\right)}^{\prime }H_{i}\left(\Delta y_{i}-\phi_{i}\xi_{i}\left(\theta \right)\right)\;\left(4\right) \end{array}$$

where $\;H_{i}=I_{T}-\;W_{i}{\left(W_{i}^{\prime }{}^{W_{i}}\right)}^{-1}W_{i}^{\prime };\varphi ={\left(\theta^{\prime },\;\phi^{\prime },\sigma^{\prime }\right)}^{\prime };\;\phi =(\phi_{1},\phi_{2},\;\ldots ,\;\phi_{N}{)}^{\prime };\text{ and }\sigma =(\sigma_{1}^{2},\sigma_{2}^{2},\ldots ,\sigma_{N}^{2}{)}^{\prime }$.

The maximum likelihood (ML) estimation of long-run coefficients, **θ**, and group-specific error-correction coefficients ϕ_*i*_, can be computed by optimizing (Equation 4) with respect to **φ**. These ML estimators for reference will be PMG estimators in order to highlight both the pooling implied by homogeneity restrictions on long-run coefficients and averaging across groups used to derive means of the estimated error-correction coefficients and other short-run parameters of the model.

We assume our equation for income elasticity of healthcare spending as,


$$\begin{array}{l} he_{it}=\theta_{0i}+\;\theta_{1i}y_{it}+\theta_{2i}le_{it}+\;\theta_{3i}oop_{it}+\mu_{i}+\epsilon_{it},\\ i=1,2,\ldots ..,N\;,\;t=1,2,\ldots ,T\;\left(5\right) \end{array}$$

Where, number of countries, *i* = 1, 2, …, *N*; number of periods, *t* = 1, 2, …, *T*; *he*_*it*_ is the log of CHE per capita; *y*_*it*_ is GDP per capita; *le*_*it*_ is LE at birth; and *oop*_*it*_ is OOP (% of CHE). The choice of right-side variables, especially the control variables, are determined by the variables used in various studies as well as availability.

Now, if the variables are *I*(1) and cointegrated, then the error term is *I*(0) for all *i*. The *ARDL* (1,1,1) dynamic panel specification of Equation (5) is, therefore,


$$\begin{array}{l} he_{it}=\delta_{10i}y_{it}+\delta_{11i}y_{i,t-1}+\delta_{20i}le_{it}+\delta_{21i}le_{i,t-1}+\delta_{30i}\;oop_{it}\\ +\delta_{31i}\;oop_{i,t-1}+\lambda_{i}he_{i,t-1}\mu_{i}+\epsilon_{it} \end{array}$$

And the error correction equation is,


$$\begin{array}{l} \Delta he_{it}=\phi_{i}\left(he_{i,t-1}-\theta_{0i}-\theta_{1i}y_{it}-\theta_{2i}le_{it}-\;\theta_{3i}oop_{it}\right)-\delta_{11i}\Delta y_{it}\\ -\delta_{21i}\Delta le_{it}-\delta_{31i}\;\Delta {oop}_{it\;+}\epsilon_{it} \end{array}$$

Where, ${\;\phi }_{i}=-\left(1-\lambda_{i}\right);\;\theta_{0i}=\frac{\mu_{i}}{1-\lambda_{i}};\;\theta_{1i}=\frac{\delta_{10i}+\delta_{11i}}{1-\lambda_{i}};\;\theta_{2i}=\frac{\delta_{20i}+\delta_{21i}}{1-\lambda_{i}};\;\theta_{3i}=\frac{\delta_{30i}+\delta_{31i}}{1-\;\lambda_{i}}$.

The error-correction speed of the adjustment parameter, ϕ_*i*_ and long-run coefficients, θ_1*i*_, θ_2*i*_, and θ_3*i*_, are of our prime concern. A non-zero mean of a cointegrating relationship is allowed by the insertion of θ_0*i*_. We expect ϕ_*i*_ to be negative when variables expiate to long-run equilibrium.

In accordance with our estimations, we hinge on the PMG estimation method, by and large, for analysis and interpretation of the parameters. However, PMG does not give country-specific long-run estimates, we rely on MG estimates for that purpose. A similar approach is used by Anderson and Shimul (68). The main justifications for such estimates are manifold. First, long-run responses of healthcare expenditures to income and other variables are likely to be similar across countries, although short-run adjustments in healthcare spending, depending on patterns of investment in health, are unlikely to be homogeneous across countries. Again, the PMG estimator allows us to investigate long-run homogeneity without imposing parameter homogeneity in the short run. Second, as econometric theory suggests imposing homogeneity causes an upward bias in the coefficient of lagged dependent variable which makes the MG estimator inefficient since it may be sensitive to extreme values or outliers. Third, if the focus of analysis is on average (across countries) income elasticities, then PMG estimates are probably preferable to MG estimates on the grounds of their better precision. What is more, it is less sensitive to lag order used in estimation, irrespective of the sizes of *T* and *N*, in contrast to MG and DFE estimators. Fourth, one advantage of PMG over the traditional DFE model is that it can allow short-run dynamic specification to differ from country to country as the PMG model is less restrictive.

Since we are considering a wide range of countries with different time periods, heterogeneity across countries is quite expected. However, we will use a homogeneity test (69) to understand whether data exhibits heterogeneity across countries.

We prefer to estimate only the income elasticity of health spending rather than any causality estimation. In such cases, we can ignore endogeneity if prevails, analogous to Anderson and Shimul (68), Pesaran et al. (25), and Pesaran et al. (66). More specifically, we use the PMG method to estimate short-run elasticities and error corrections across countries. Error correction close to one indicates that it can recover healthcare spending from GDP shocks straight away. We rely on MG estimators for long-run elasticity estimates. Since the PMG method constrains long-run coefficients to be equal across groups, the MG estimator is a simple arithmetic average of the coefficients, which can be calculated separately for each group.

## 4. Findings and discussions

We report the estimates of elasticities in this section using dynamic heterogeneous panel estimators PMG, and MG, along with DFE estimates. As mentioned earlier, for long-run overall estimates we rely on PMG estimation.^2^ However, we report MG estimates for long-run country-specific estimates. In addition, we record DFE estimates for comparisons.

In addition, the homogeneity test (69) suggests that the data used here are heterogeneous as Delta Statistic is statistically significant (*p* = 0.000) (see Supplementary Table A1).

Table 1 reports the long-run estimates of PMG, MG, and DFE estimators for GDP per capita, LE at birth, and OOP (% of CHE). The preferred PMG long-run elasticity is 1.051 which is significantly different from zero but not much different from unity. The elasticity of more than one indicates that current healthcare expenditure per capita changes more than proportionately with changes in the GDP of the country. This finding is analogous to Bhat and Jain (14), Liu et al. (16), Hitiris (37), Wang and Rettenmaier (15), Newhouse (2), Cullis and West (4), Moore et al. (12), Culyer (13), Maxwell (5), Kleiman (3), Gertler and Van der Gaag (6), Clemente et al. (23), Mehrara et al. (36), and Fedeli (65). PMG estimates show that LE at birth and OOP also have a significant long-run relationship with CHE per capita. Short-run PMG estimates of elasticities are not significantly different from zero. The short-run error correction parameter is −0.295, indicating a 30% correction (in opposite direction) in the first year following a country's GDP shock. After 3 years, 90% of the disequilibrium is removed and in the fourth year, all the disequilibrium is recovered. Consequently, the speed of adjustment of GDP shock is relatively moderate.

**Table 1:** Panel regression estimates of elasticities.

**Log of Current Health Expenditure per capita**	**(1) MG**	**(2) PMG**	**(3) DFE**
**Long-run estimates**			
Log of GDP per capita	0.201 (0.240)	1.051^***^ (0.022)	0.954^***^ (0.045)
Log of life expectancy at birth	5.901^***^ (1.960)	−2.695^***^ (0.186)	−0.276 (0.318)
Out-of-pocket expenditure (% of current health expenditure)	−0.060 (0.054)	−0.004^***^ (0.001)	−0.005^***^ (0.002)
**Short-run estimates**			
Error correction	−0.755^***^ (0.033)	−0.295^***^ (0.023)	−0.240^***^ (0.012)
Change in log of GDP per capita	−0.093 (0.101)	0.105 (0.083)	0.203^***^ (0.040)
Change in log of life expectancy at birth	29.13 (26.27)	−5.380 (5.091)	0.793^*^ (0.447)
Change in out-of-pocket expenditure (% of current health expenditure)	0.015 (0.016)	−0.047 (0.036)	−0.003^***^ (0.001)
Constant	−7.134 (5.709)	2.457^***^ (0.209)	−0.249 (0.278)
Observations	2,790	2,790	2,790

Standard errors in parentheses and ^*^p < 0.10, ^**^p < 0.05, ^***^p < 0.01.

MG estimates indicate a long-run elasticity of 0.201 which is not significantly different from zero, but it is far lower than PMG estimate. MG estimates also show that LE at birth has a significant long-run relationship with CHE per capita. Short-run error correction is −0.755 indicating a relatively strong 76% correction (in opposite direction) in the first year following a country's GDP shock. It indicates that in the second year, all the disequilibrium is removed. Hence, the speed of adjustment of GDP shock is relatively fast in this case. The short-run MG estimates of elasticities are not significantly different from zero, as same as PMG estimates. Recall that, PMG long-run estimates are identical across countries, whereas MG estimates separate estimates for different time series of different countries and then averages those. Hence, an MG elasticity of 0.201 is the average of the country time series estimates. On the other hand, PMG estimates allow short-run estimates to vary across countries but constrains long-run estimates to be identical. Hence, the PMG estimate of 1.051 shows the common long-run elasticity for all 177 countries.

For comparison, DFE estimates are also presented in the last two columns of Table 1. It shows long-run elasticity is 0.954 and short-run error correction is −0.240. DFE long-run elasticity is bracketed by PMG and MG estimates, but DFE short-run error correction is smaller than the other two estimates. PMG estimates are preferred due to the nature of our dynamic heterogeneous panel data whereas DFE estimator estimates simply pool the cross-section data.

### 4.1. Heterogeneity and country-specific estimates of elasticities

Along with long-run and short-run common estimates, we provide the country-specific estimates considering heterogeneity. This is to be noted that those countries with at least one of the three (long-run, short-run, and short-run error correction) estimates significantly different from zero are presented in Table 2.^3^ Even though PMG is the preferred method for aggregate level results, we relied on MG estimation for long-run elasticities when we intend to understand differences in long-term elasticities across countries. In addition, we relied on the PMG estimation for short-run elasticities. Column (1) presents long-run elasticities, column (2) shows short-run elasticities, and column (3) gives short-run error correction estimates.

**Table 2:** PMG elasticity error correction estimates.

**Country name**	**(1) Long-run**	**(2) Short-run**	**(3) Short-run error correction**
Angola	2.024^***^	−1.670^***^	−1.220^***^
Australia	1.417^*^	0.345^**^	−0.290^***^
Austria	1.055^*^	0.400^**^	−0.111^**^
Bosnia and Herzegovina	2.353^***^	0.973^***^	−0.191^**^
Canada	1.115^**^	−0.288^***^	−0.199^***^
Spain	0.729^***^	0.336^***^	−0.082^***^
France	0.883^*^	0.438^**^	−0.071^*^
Georgia	1.249^***^	−0.534^**^	−0.489^***^
Ireland	−0.201^**^	−0.217^***^	−0.150^***^
Israel	1.390^***^	0.570^***^	−0.371^**^
Cambodia	−0.655^**^	−1.098^*^	−0.532^***^
Portugal	1.606^***^	0.393^*^	−0.179^***^
Sierra Leone	0.885^***^	−2.100^***^	−1.996^***^
Serbia	1.770^***^	1.042^***^	−0.169^**^
Seychelles	2.417^***^	0.867^*^	−0.631^***^
Tanzania	3.065^**^	3.365^***^	−0.560^***^
Uganda	−20.64	2.869^**^	−0.219^***^
Yemen, Rep.	1.355^***^	1.022^***^	−0.273^**^

^*^p < 0.10, ^**^p < 0.05, ^***^p < 0.01.

Long-run MG elasticity estimates are reported in column (1), as the long-run PMG estimator constrains estimates to be identical for each country. MG estimates are significantly different from zero for 78 countries. Though Table 1 reports the average MG long-run coefficient as 0.201, a wide range (−8.291 to 4.174) of estimates is evident here specifying substantial heterogeneity of data. Of the 78 countries, 50 have estimates above the average (0.915) and 47 have estimated coefficients of more than one, indicating more than proportionate changes in CHE per capita with shocks in GDP of the country. Interestingly, countries with high GDP per capita (i.e., developed countries) have estimated coefficients of more than one including Australia, Austria, Canada, Estonia, Finland, the United Kingdom, Israel, Italy, Lithuania, Latvia, Malaysia, Poland, Portugal, Slovak Republic, and Tanzania. Fogel (20), Hitiris (37), Okunade and Murthy (19), Bhat and Jain (14), Clemente et al. (23), Liu et al. (16), and Wang and Rettenmaier (15) also found similar finding of more than unitary elasticity in developed countries in long-run. Hence, countries with relatively lower GDP per capita have a long-run elasticity of less than one which goes in line with Kea et al. (46), Farag et al. (47), Zare et al. (48), Lv and Zhu (49), Bustamante and Shimoga (50), and Rana et al. (60). Baltagi and Moscone (55), Mehrara et al. (70), and Abdullah et al. (52) also observe similar findings for developed countries. Iheoma (64) also uses the PMG which restricts the long-run estimates to be equal across countries, while the short-run relationship captures country-specific heterogeneity. The other way around, the MG estimator allows for heterogeneity in the short and long-run relationships between economic uncertainty and health expenditure per capita.

Short-run PMG elasticity estimates are reported in column (2). PMG estimates are significantly different from zero for 58 countries. Rest (119) countries do not respond to changes in GDP in the short run. Though Table 1 reports the average PMG short-run coefficient as 1.051, a wide range (−8.049 to 3.365) of estimates is evident here also showing substantial heterogeneity of data. Of the 58 countries, 35 have estimates above the average (0.048) and 16 countries have estimated coefficients of more than one. Countries with relatively lower GDP per capita have estimated short-run elasticity of more than one including Bangladesh, Brazil, Honduras, Jordan, Pakistan, Paraguay, Serbia, Uganda, and the Republic of Yemen. Tanzania is an exception in this case. Our study found that developed countries have a short-run elasticity of less than one which endorses (71).

In column (3), short-run error correction estimates of 108 countries that are significantly different from zero are reported. This column also shows heterogeneity across countries in the process of error correction.

**Figure 1:**
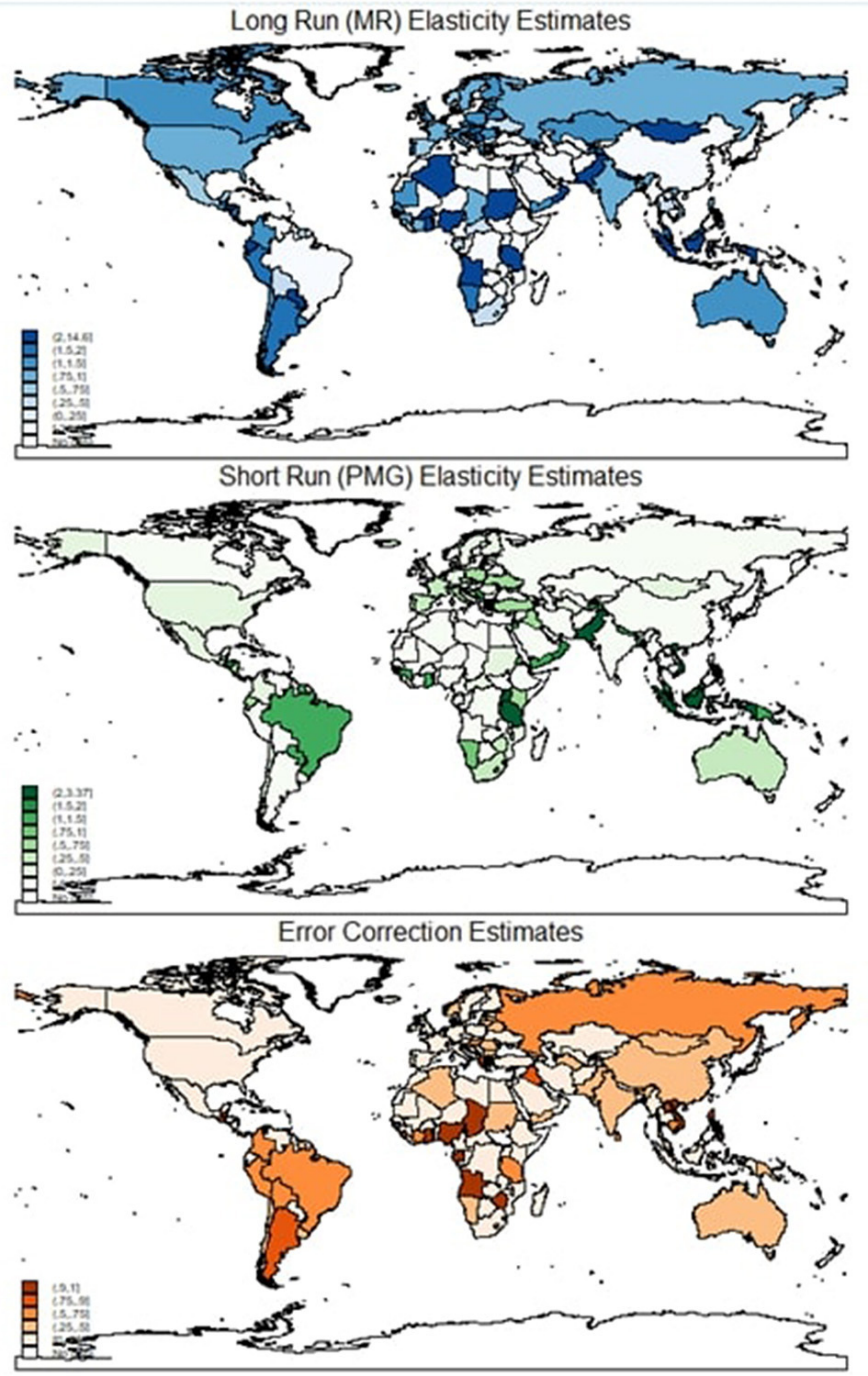
Elasticity estimates.

Though Table 1 reports the average short-run error correction coefficient as 0.954, a wide range of estimates is evident here varying from −0.996 to 0.489, showing considerable heterogeneity. Of the 50 countries with slow error correction process (estimates are about 0.30 or less), Spain, France, Gambia, and Oman have the slowest error correction process (estimates are about 0.1 or less). Ninety countries have moderate error correction processes (estimates are more than 0.50 and < 0.75), and 18 countries have fast error correction countries (estimates are at least 0.75). Of these, Angola, Gabon, Nigeria, Philippines, Sierra Leone, Chad, Vietnam, and Zimbabwe have the fastest error correction process (estimated coefficients of at least 1). Hence, column (3) reveals extensive heterogeneity in country GDP which is revealed by using dynamic panel estimators. Figure 1 depicts the elasticity estimates across the globe. It is clear that the long-run elasticities of most countries, except for a few African nations, are quite large. But this is not quite true for short-run estimates as there is a large variation in short-run estimates.

Even though short-run estimates are not statistically different from zero for many countries, error correction coefficients are significant. This phenomenon indicates that the elasticities of most countries are driven by long-run behavior rather than short-run one and countries revert to long-run equilibrium quickly once there is an income shock.

Table 3 reports descriptive properties of the estimates and shows heterogeneity in estimates across countries. There is substantial variation in the long-run, as shown by the large (16.053) coefficient of variation (CV). Long-run estimates ranged from a minimum of −20.64 to a maximum of 14.59, whereas the mean and median estimates are 0.201 and 0.796, respectively. The 25th and 75th percentiles are similarly very widely divergent, indicating substantial variation in the long-run experience of the countries.

**Table 3:** Properties of elasticity estimates.

**Estimate type**	**Mean**	**SD**	**Min**	**Max**	**25th percentile**	**50th percentile**	**75th percentile**	**CV**
Long-run	0.201	3.233	−20.64	14.59	−0.182	0.796	1.488	16.053
Short-run	0.105	1.110	−8.049	3.365	−0.312	0.141	0.591	10.562
Error correction	−0.295	0.337	−1.996	0.489	−0.421	−0.186	−0.105	−1.140

Long-run estimates are MG estimates. Short-run and error correction estimates are PMG estimates.

Short-run estimates (mean 0.105 and median 0.141) also show a wide variation in the experience of countries, as indicated by large CV (10.562). Error correction process (mean −0.295 and median 0.141) shows limited variation is found in the case of the error correction process, as indicated by a small CV. It is also found that there is a positive correlation between short-run estimates and error correction estimates (correlation coefficient is 0.26 with a *p*-value < 0.01), indicating countries with significant and faster error correction processes have larger short-run estimates.

We also tested for cross-sectional dependency (CD) across panels using Pesaran's (72) CD test for weak cross-sectional dependence. The CD statistic is −0.702 which is statistically insignificant (*p* = 0.483). Thus, cross sectional dependency is not an issue for this study. We tested stationarity where GDP per capita (log of GDP per capita) was found non-stationary in level form (see Supplementary Table A5). However, as PMG can incorporate non-stationary variables, our estimates are suitable for heterogeneous non-stationary panels (73) as well.

One limitation of this study is worth noting. With long time series, the chance of having infrequent shocks that leave a permanent effect on a variable is high. Since this study includes panel data with long time series, structural breaks are not unlikely. However, the current study did not include a structural break in the estimation, partly because the structural break issue is more important for time series data and the option of using a structural break with PMG is limited, if not irrelevant. Another limitation of the study is that it is mostly an empirical exercise without using any explicit theoretical model. However, similar approaches are not uncommon in this type of study in literature, such as in Dogan et al. (74), Fedeli (65), Mehmood et al. (62), and Iheoma (64). In addition, no explicit political propositions are considered in the regression, nor did we include a regional analysis. However, in our analysis, we attempted to understand the differences across countries.

## 5. Conclusion and policy recommendations

Our analysis aimed at estimating both short-run and long-run responsiveness of healthcare spending to changes in a country's GDP using suitable statistical tools for non-stationary dynamic heterogeneous panels. For this purpose, our analysis includes estimates of short-run, error correction, and long-run responses using MG, PMG, and DFE estimators. These estimators have not been used in health economics literature to date and are conventionally suited to the heterogeneous experience of 177 countries over 60 years of analyses. Using MG and PMG estimates, we determine the heterogeneity in the elasticities of healthcare spending.

Based on our preferred PMG estimation method, healthcare spending is responsive when a country's GDP changes, with an estimated elasticity in excess of unity: 1.051. Positive GDP shocks result in more than proportional changes in healthcare spending, whereas negative shocks end in larger reductions. Though healthcare spending is more sensitive to changes in state GDP in long run, the error correction process is relatively prolonged. The error correction term is only −0.295, indicating that healthcare spending recovers only 30% of that change in the following year for a country's GDP shock. Furthermore, long-run MG elasticity estimates reveal that, even though the overall estimated elasticity is very low (0.201), the error correction process is rapid with the value of −0.755, indicating that in the second year, all the disequilibrium is removed.

The majority (90 out of 108) countries have moderate error correction processes with estimates of more than 0.50 and < 0.75. Of them, eight have the fastest error correction process with estimated coefficients close to 1. From country-specific estimation, it was revealed that developed countries have estimated long-run elasticity of more than one and less developed countries have estimated short-run elasticity of more than one, indicating that developed countries' healthcare spending is responsive to GDP shock in long-run whereas least developed country are responsive in short-run. Now, these findings have enormous implications for developing countries as most countries of the world are now facing COVID-19 and post-COVID-19. Since many developing countries' estimated short-run elasticity is relatively larger, developing countries may witness more fluctuation in their healthcare expenditure due to GDP shocks which are expected to occur in near future. This shock in healthcare expenditure may have a negative effect on the population. Therefore, countries should pay sincere attention to keeping their healthcare expenditure stable. This study provides some guidance on how countries will revert to their long-run trend of healthcare expenditure based on their historical trend. It is worth mentioning that even though the current study provides robust estimates, some of its limitations such as reliance on mostly empirical analysis without strong theoretical justification may plague the findings. Future research can address those limitations to improve the estimates.

## Data availability statement

The original contributions presented in the study are included in the article/Supplementary material, further inquiries can be directed to the corresponding author.

## Author contributions

SNS conceptualized the study and analysis plan, analyzed the data, and finalized the manuscript. MIUK analyzed the data and was a major contributor to writing the draft. FK reviewed the related literature and was a major contributor to writing the draft. All authors read and approved the final manuscript.

## Footnotes

^1^The current study in fact estimates buoyancy rather than elasticity. But the term “buoyancy” is not common in economics literature, so we use elasticity.

^2^It is worth mentioning that in choosing PMG vs MG for long run coefficients, we performed Hausman test (see Supplementary Table A2) and we found that PMG is preferred estimation method in eliciting long run coefficients. In addition, healthcare expenditure and GDP are cointegrated as Westerlund ECM panel cointegration tests cannot the reject the null of no cointegration by all three statistics out of four (see Supplementary Table A3).

^3^Table 2 presents only the countries having all the three significant estimates to various level (1, 5, or 10%). The detailed long-run, short-run elasticity estimates and short-run error correction estimates of all 177 countries are in Supplementary Table A4.
