# Supplementary material for: Resiliency of healthcare expenditure to income shock: Evidence from dynamic heterogeneous panels
Source: Front Public Health. 2023 Mar 7;11:1085338. doi: 10.3389/fpubh.2023.1085338 (PMC10027743; doi:10.3389/fpubh.2023.1085338)
Supplement: Supplementary file 1 [file Table_1.docx]

**Appendix**

*[Insert ‘Table A1: Testing for homogeneity’ here]*

*[Insert ‘Table A2: Hausman Test’ here]*

*[Insert ‘Table A3: Westerlund ECM panel cointegration tests’ here]*

*[Insert ‘Table A4: PMG Elasticity Error Correction Estimates’ here]*

*[Insert ‘Table A5: Stationarity test (Unit root test)’ here]*

**Table 1: Panel Regression Estimates of Elasticities**

| **Log of Current Health Expenditure per capita** | | | **(1)**  **MG (without structural breaks)** | **(2)**  **MG**  **(with structural breaks)** | **(3)**  **PMG (without structural breaks)** | **(4)**  **PMG**  **(with structural breaks)** | **(5)**  **DFE (without structural breaks)** | **(6)**  **DFE**  **(with structural breaks)** |
| --- | --- | --- | --- | --- | --- | --- | --- | --- |
|  |  | **Long-run estimates** | | | | | |  |
| Log of GDP per capita | | | 0.201 (0.240) |  | 1.051^***^ (0.022) |  | 0.954^***^ (0.045) |  |
| Log of life expectancy at birth | | | 5.901^***^ (1.960) |  | -2.695^***^ (0.186) |  | -0.276 (0.318) |  |
| Out-of-pocket expenditure  (% of current health expenditure) | | | -0.060 (0.054) |  | -0.004^***^ (0.001) |  | -0.005^***^ (0.002) |  |
|  |  | **Short-run estimates** | | | | | |  |
| Error correction | | | -0.755^***^ (0.033) |  | -0.295^***^ (0.023) |  | -0.240^***^ (0.012) |  |
| Change in log of GDP per capita | | | -0.093 (0.101) |  | 0.105 (0.083) |  | 0.203^***^ (0.040) |  |
| Change in log of life expectancy at birth | | | 29.13 (26.27) |  | -5.380 (5.091) |  | 0.793^*^ (0.447) |  |
| Change in out-of-pocket expenditure  (% of current health expenditure) | | | 0.015 (0.016) |  | -0.047 (0.036) |  | -0.003^***^ (0.001) |  |
| Constant | | | -7.134 (5.709) |  | 2.457^***^ (0.209) |  | -0.249 (0.278) |  |
| Observations | | | 2,790 |  | 2,790 |  |  |  |

Notes: Standard errors in parentheses and ^*^ *p* < .10, ^**^ *p* < .05, ^***^ *p* < .01

**Table 2: PMG Elasticity Error Correction Estimates**

| **Country Name** | **(1)**  **Long-run** | **(2)**  **Short-run** | **(3)**  **Short-run error correction** |
| --- | --- | --- | --- |
| Angola | 2.024^***^ | -1.670^***^ | -1.220^***^ |
| Australia | 1.417^*^ | 0.345^**^ | -0.290^***^ |
| Austria | 1.055^*^ | 0.400^**^ | -0.111^**^ |
| Bosnia and Herzegovina | 2.353^***^ | 0.973^***^ | -0.191^**^ |
| Canada | 1.115^**^ | -0.288^***^ | -0.199^***^ |
| Spain | 0.729^***^ | 0.336^***^ | -0.082^***^ |
| France | 0.883^*^ | 0.438^**^ | -0.071^*^ |
| Georgia | 1.249^***^ | -0.534^**^ | -0.489^***^ |
| Ireland | -0.201^**^ | -0.217^***^ | -0.150^***^ |
| Israel | 1.390^***^ | 0.570^***^ | -0.371^**^ |
| Cambodia | -0.655^**^ | -1.098^*^ | -0.532^***^ |
| Portugal | 1.606^***^ | 0.393^*^ | -0.179^***^ |
| Sierra Leone | 0.885^***^ | -2.100^***^ | -1.996^***^ |
| Serbia | 1.770^***^ | 1.042^***^ | -0.169^**^ |
| Seychelles | 2.417^***^ | 0.867^*^ | -0.631^***^ |
| Tanzania | 3.065^**^ | 3.365^***^ | -0.560^***^ |
| Uganda | -20.64 | 2.869^**^ | -0.219^***^ |
| Yemen, Rep. | 1.355^***^ | 1.022^***^ | -0.273^**^ |

Notes: ^*^ *p* < .10, ^**^ *p* < .05, ^***^ *p* < .01

**Table 3: Properties of Elasticity Estimates**

| **Estimate Type** | **Mean** | **SD** | **Min** | **Max** | **25^th^ Percentile** | **50^th^ Percentile** | **75^th^ Percentile** | **CV** |
| --- | --- | --- | --- | --- | --- | --- | --- | --- |
| Long-run | 0.201 | 3.233 | -20.64 | 14.59 | -0.182 | 0.796 | 1.488 | 16.053 |
| Short-run | 0.105 | 1.110 | -8.049 | 3.365 | -0.312 | 0.141 | 0.591 | 10.562 |
| Error correction | -0.295 | 0.337 | -1.996 | 0.489 | -0.421 | -0.186 | -0.105 | -1.140 |

Note: Long-run estimates are MG estimates. Short-run and error correction estimates are PMG estimates

**Table A1: Testing for homogeneity**

H_0_: slope coefficients are homogenous

H_A_: slope coefficients are heterogeneous

|  | Delta | p-value |
| --- | --- | --- |
|  | 14.515 | 0.000 |
| adj. | 17.566 | 0.000 |

HAC Kernel: bartlett

with average bandwith 1.9378531

Variables partialled out: constant

**Table A2: Hausman Test**

| Variable | Coefficients | |  |  |
| --- | --- | --- | --- | --- |
|  | (b) | (B) | (b-B) | SE |
|  | MG | PMG | Difference | 0.524886 |
| LnGDP | 0.201418 | 1.051283 | -0.84986 | 4.236384 |
| Ln of Life Exp. | 5.900856 | -2.69462 | 8.595475 | 0.111092 |
| Out of Pocket Payment | -0.06007 | -0.00387 | -0.0562 |  |
| $\chi_{(2)}^{2}=$5.33  Prob>$\chi_{(2)}^{2}$ = 0.1492 | | | | |

**Table A3: Westerlund ECM panel cointegration tests**

| Statistic | Value | Z-value | P-value |
| --- | --- | --- | --- |
| Gt | -3.386 | -17.304 | 0.000 |
| Ga | -18.598 | -13.588 | 0.000 |
| Pt | -26.079 | 2.824 | 0.998 |
| Pa | -11.890 | -6.632 | 0.000 |

**Table A4: PMG Elasticity Error Correction Estimates**

| **Sl** | **Country Name** | **(1)**  **Long-run** | **(2)**  **Short-Run** | **(3)**  **Short-run error correction** |
| --- | --- | --- | --- | --- |
| 1 | Afghanistan | -0.280 | -0.339 | -0.388 |
| 2 | Angola | 2.024^***^ | -1.670^***^ | -1.220^***^ |
| 3 | Albania | 0.229 | 0.133 | -0.128 |
| 4 | United Arab Emirates | -0.590 | -0.156 | -0.016 |
| 5 | Argentina | 1.668^***^ | -0.343 | -0.845^***^ |
| 6 | Armenia | 0.961^***^ | 0.488 | 0.012 |
| 7 | Antigua and Barbuda | 0.679^***^ | 0.141 | -0.957^***^ |
| 8 | Australia | 1.417^*^ | 0.345^**^ | -0.290^***^ |
| 9 | Austria | 1.055^*^ | 0.400^**^ | -0.111^**^ |
| 10 | Azerbaijan | -1.038 | -0.243 | -0.178 |
| 11 | Burundi | -16.44 | 0.970 | -0.180^*^ |
| 12 | Belgium | 1.799 | -0.509^*^ | -0.109^***^ |
| 13 | Benin | -1.012 | -0.455 | -0.239 |
| 14 | Burkina Faso | 2.488^*^ | -0.957 | -0.040 |
| 15 | Bangladesh | 0.502 | 1.119^**^ | -0.024 |
| 16 | Bulgaria | 0.466^***^ | -0.405 | -0.276^***^ |
| 17 | Bahrain | 0.603 | 0.325 | -0.384^**^ |
| 18 | Bahamas | -0.139 | -0.197 | -0.235^**^ |
| 19 | Bosnia and Herzegovina | 2.353^***^ | 0.973^***^ | -0.191^**^ |
| 20 | Belarus | 1.449^***^ | -0.099 | -0.340^*^ |
| 21 | Belize | 0.145 | -0.572 | -0.036 |
| 22 | Bolivia | 0.420 | -2.002^***^ | -0.714^***^ |
| 23 | Brazil | -0.182 | 1.145^***^ | -0.548^***^ |
| 24 | Barbados | -0.247 | 0.116 | -0.127 |
| 25 | Brunei Darussalam | -3.181 | -2.292^***^ | -0.521^***^ |
| 26 | Bhutan | 1.666^***^ | -0.296 | -0.644^***^ |
| 27 | Botswana | -0.614 | -0.648^**^ | 0.017 |
| 28 | Central African Republic | 0.289^***^ | 0.110 | -0.139^**^ |
| 29 | Canada | 1.115^**^ | -0.288^***^ | -0.199^***^ |
| 30 | Switzerland | 1.758 | 0.175 | -0.079 |
| 31 | Chile | 1.220 | 0.001 | -0.255^***^ |
| 32 | China | -0.159 | -1.314^***^ | -0.321^***^ |
| 33 | Cote d'Ivoire | 0.963^**^ | -0.267 | -0.709^***^ |
| 34 | Cameroon | -0.701 | -1.323^**^ | 0.050 |
| 35 | Democratic Republic of the Congo | 2.223 | 0.626 | -0.402^***^ |
| 36 | Republic of the Congo | -3.724 | -2.517^***^ | 0.008 |
| 37 | Colombia | 1.028 | 0.163 | -0.510^***^ |
| 38 | Comoros | 3.946 | 0.688 | 0.006 |
| 39 | Cabo Verde | -0.021 | -0.407 | -0.161 |
| 40 | Costa Rica | 1.604^***^ | 0.682^**^ | -0.129 |
| 41 | Cyprus | 0.885^***^ | 0.436 | -0.394^***^ |
| 42 | Czech Republic | 1.340^**^ | 0.436 | -0.207^**^ |
| 43 | Germany | -1.599 | 0.066 | -0.114^**^ |
| 44 | Denmark | 2.075^**^ | 0.249 | -0.129^***^ |
| 45 | Dominican Republic | 0.103 | 0.394 | -0.326^**^ |
| 46 | Algeria | 3.334 | -1.813^*^ | -0.421^**^ |
| 47 | Ecuador | 5.452 | 0.537 | -0.154^**^ |
| 48 | Egypt | -0.035 | 0.537 | 0.209 |
| 49 | Eritrea | 0.662 | 0.753^*^ | -0.020 |
| 50 | Spain | 0.729^***^ | 0.336^***^ | -0.082^***^ |
| 51 | Estonia | 1.295^***^ | 0.092 | -0.142^**^ |
| 52 | Ethiopia | 0.822 | 0.698 | -0.202 |
| 53 | Finland | 1.066^***^ | 0.156 | -0.122^***^ |
| 54 | Fiji | 1.488^**^ | 0.249 | -0.685^***^ |
| 55 | France | 0.883^*^ | 0.438^**^ | -0.071^*^ |
| 56 | Federal States of Micronesia | -0.678 | 1.171^***^ | -0.225^***^ |
| 57 | Gabon | 0.313 | -2.032^*^ | -1.132^***^ |
| 58 | United Kingdom | 1.402^***^ | 0.001 | -0.149^***^ |
| 59 | Georgia | 1.249^***^ | -0.534^**^ | -0.489^***^ |
| 60 | Ghana | 2.070 | 1.269 | -0.943^***^ |
| 61 | Guinea | 0.814 | 1.211 | -0.214^*^ |
| 62 | Gambia | 1.761 | -0.150 | 0.489^***^ |
| 63 | Guinea-Bissau | -6.469 | -0.175 | 0.002 |
| 64 | Equatorial Guinea | -0.619 | 0.520^*^ | -0.045 |
| 65 | Greece | 1.092 | -0.523^***^ | -0.830^***^ |
| 66 | Grenada | 0.638^***^ | -0.136 | -0.481^***^ |
| 67 | Guatemala | 1.147^***^ | 0.300 | -0.933^***^ |
| 68 | Guyana | 1.227 | 0.395 | -0.047 |
| 69 | Honduras | 3.240 | 1.740^***^ | -0.084 |
| 70 | Croatia | 1.857^***^ | 0.143 | -0.349^**^ |
| 71 | Haiti | -1.374^*^ | 0.274 | 0.087 |
| 72 | Hungary | 1.958 | -0.146 | -0.571^***^ |
| 73 | Indonesia | 2.768 | 3.056 | -0.159^*^ |
| 74 | India | 0.919 | -0.130 | -0.367^***^ |
| 75 | Ireland | -0.201^**^ | -0.217^***^ | -0.150^***^ |
| 76 | Iran | 0.523 | 0.379 | -0.081 |
| 77 | Iraq | -2.317 | 0.591 | -0.861^***^ |
| 78 | Iceland | 0.659^**^ | 0.319^**^ | -0.140 |
| 79 | Israel | 1.390^***^ | 0.570^***^ | -0.371^**^ |
| 80 | Italy | 1.034^***^ | 0.506^**^ | -0.098 |
| 81 | Jamaica | 1.525^***^ | -0.837 | -0.806^***^ |
| 82 | Jordan | 0.796 | 1.018^*^ | -0.197 |
| 83 | Japan | -0.235 | -0.073 | -0.007 |
| 84 | Kazakhstan | 1.163^*^ | -1.307 | -0.131 |
| 85 | Kenya | -1.557 | 0.595^**^ | -0.142^***^ |
| 86 | Kyrgyz Republic | 1.581 | 0.196 | -0.147 |
| 87 | Cambodia | -0.655^**^ | -1.098^*^ | -0.532^***^ |
| 88 | Kiribati | 0.712 | 0.643 | -0.190 |
| 89 | Republic of Korea | 0.652 | 0.394 | -0.041 |
| 90 | Kuwait | 0.642 | -1.079^**^ | -0.218 |
| 91 | Lao PDR | -3.937 | -8.049^***^ | -0.771^***^ |
| 92 | Lebanon | 0.763 | 0.191 | -0.512^***^ |
| 93 | Liberia | -0.665^*^ | 0.805 | 0.014 |
| 94 | Libya | 0.683 | 0.055 | -0.253 |
| 95 | Saint Lucia | 0.373 | 0.074 | -0.453^**^ |
| 96 | Sri Lanka | 0.447^***^ | -0.582 | -0.557^***^ |
| 97 | Lesotho | -0.675^**^ | 0.052 | -0.150^**^ |
| 98 | Lithuania | 1.067^***^ | 0.121 | -0.292^***^ |
| 99 | Luxembourg | -0.135 | -0.312 | -0.426^***^ |
| 100 | Latvia | 1.113^***^ | 0.092 | -0.483^*^ |
| 101 | Morocco | -1.582^***^ | -0.316 | -0.047 |
| 102 | Moldova | 2.218^***^ | -0.425 | -0.307^***^ |
| 103 | Madagascar | 0.849 | 0.138 | -0.291 |
| 104 | Maldives | 0.543 | -0.276 | -0.861^***^ |
| 105 | Mexico | 0.694^**^ | 0.175 | 0.143 |
| 106 | North Macedonia | 0.158 | 1.019^*^ | -0.096 |
| 107 | Mali | -5.264^***^ | -1.242^**^ | -0.175 |
| 108 | Malta | -5.777 | 0.495 | -0.159^**^ |
| 109 | Myanmar | -1.353 | 1.401 | -0.107 |
| 110 | Mongolia | 2.605 | 0.236 | -0.495^**^ |
| 111 | Mozambique | -1.095 | 0.161 | -0.240^*^ |
| 112 | Mauritania | 1.059^**^ | -0.510 | -0.106 |
| 113 | Mauritius | 1.075 | -0.832 | -0.015 |
| 114 | Malawi | 1.862 | 2.264 | -0.029 |
| 115 | Malaysia | 1.593^**^ | 1.834^***^ | 0.164 |
| 116 | North America | 0.834^***^ | 0.106 | -0.144^***^ |
| 117 | Namibia | 1.512^**^ | 0.850 | -0.287^*^ |
| 118 | Niger | -1.518 | -0.174 | -0.082 |
| 119 | Nigeria | 2.105 | -1.697 | -1.109^***^ |
| 120 | Nicaragua | 1.826^***^ | 0.616 | -0.181^*^ |
| 121 | Netherlands | 2.046 | 0.231 | -0.162^***^ |
| 122 | Norway | 0.512^***^ | -0.147 | -0.297^***^ |
| 123 | Nepal | 2.001^*^ | 1.066 | -0.086 |
| 124 | New Zealand | 0.602 | 0.448 | -0.097 |
| 125 | Oman | 14.59 | 1.622 | 0.375^*^ |
| 126 | Pakistan | 4.174^***^ | 2.288^*^ | -0.275 |
| 127 | Panama | 0.754^***^ | 0.156 | -0.554^**^ |
| 128 | Peru | 1.574 | -0.622^**^ | -0.662^***^ |
| 129 | Philippines | 0.859^**^ | -0.381 | -1.359^***^ |
| 130 | Papua New Guinea | -3.949^**^ | 1.035 | -0.452^***^ |
| 131 | Poland | 1.125^***^ | 0.673^**^ | -0.164 |
| 132 | Portugal | 1.606^***^ | 0.393^*^ | -0.179^***^ |
| 133 | Paraguay | 2.176^***^ | 1.426^***^ | -0.036 |
| 134 | Qatar | -8.291^**^ | -0.459 | -0.135 |
| 135 | Romania | 1.283^***^ | 0.134 | -0.492^***^ |
| 136 | Russian Federation | 0.904^***^ | -0.241 | -0.654^***^ |
| 137 | Rwanda | -9.315 | -1.366 | -0.164^**^ |
| 138 | Saudi Arabia | -1.239 | -1.210^*^ | -0.117 |
| 139 | Sudan | 2.753^**^ | 0.063 | -0.317^**^ |
| 140 | Senegal | 1.349 | -0.281 | -0.178^*^ |
| 141 | Singapore | 3.136 | -0.172 | 0.005 |
| 142 | Solomon Islands | -1.125 | 0.772^*^ | -0.242^*^ |
| 143 | Sierra Leone | 0.885^***^ | -2.100^***^ | -1.996^***^ |
| 144 | El Salvador | -0.418 | -0.755^***^ | -0.169 |
| 145 | Serbia | 1.770^***^ | 1.042^***^ | -0.169^**^ |
| 146 | Sao Tome and Principe | -6.602 | -0.825 | 0.033 |
| 147 | Suriname | 0.744 | 1.374^***^ | -0.277^***^ |
| 148 | Slovak Republic | 1.506^***^ | 0.617^**^ | -0.075 |
| 149 | Slovenia | 1.199 | 0.118 | -0.200^***^ |
| 150 | Sweden | 0.910^***^ | 0.136 | -0.148 |
| 151 | Eswatini | -0.097 | 0.434 | -0.105^**^ |
| 152 | Seychelles | 2.417^***^ | 0.867^*^ | -0.631^***^ |
| 153 | Chad | 0.970^*^ | -0.584 | -1.278^***^ |
| 154 | Togo | 0.436 | 1.734 | -0.123 |
| 155 | Thailand | 0.385 | -0.385 | -0.186^*^ |
| 156 | Tajikistan | 1.845^***^ | 1.433^*^ | -0.024 |
| 157 | Turkmenistan | -6.144 | 0.158 | -0.276^***^ |
| 158 | Timor-Leste | 0.160 | 0.103 | -0.182^**^ |
| 159 | Tonga | -0.213 | 0.206 | -0.040 |
| 160 | Trinidad and Tobago | -0.072 | -0.791 | -0.804^***^ |
| 161 | Tunisia | 0.370 | -0.093 | -0.141^***^ |
| 162 | Turkey | -2.566 | 0.629^***^ | -0.175 |
| 163 | Tanzania | 3.065^**^ | 3.365^***^ | -0.560^***^ |
| 164 | Uganda | -20.64 | 2.869^**^ | -0.219^***^ |
| 165 | Ukraine | 0.409 | 0.694^***^ | 0.050 |
| 166 | Uruguay | 1.239^***^ | 0.008 | -0.350^**^ |
| 167 | United States | 0.796^***^ | 0.140 | -0.144^***^ |
| 168 | Uzbekistan | -0.705 | 1.223 | -0.032 |
| 169 | Saint Vincent and the Grenadines | 1.707^**^ | 0.416 | -0.410^***^ |
| 170 | Venezuela | 1.705^***^ | 0.209 | -0.494^**^ |
| 171 | Vietnam | 0.769 | 2.667^**^ | -1.043^***^ |
| 172 | Vanuatu | -1.169^***^ | -1.021^***^ | -0.194 |
| 173 | Samoa | 1.457^***^ | 0.021 | -0.446^***^ |
| 174 | Yemen | 1.355^***^ | 1.022^***^ | -0.273^**^ |
| 175 | South Africa | 0.347^**^ | 0.427 | 0.045 |
| 176 | Zambia | -13.94 | -0.680 | -0.275 |
| 177 | Zimbabwe | -0.404 | 0.500 | -1.415^***^ |

Notes: ^*^ *p* < .10, ^**^ *p* < .05, ^***^ *p* < .01

**Table A5: Stationarity test (Unit root test)**

|  | Health expenditure per capita | | Life expectancy at birth | | Out-of-pocket expenditure | | GDP per capita | |
| --- | --- | --- | --- | --- | --- | --- | --- | --- |
|  | Statistic | p-value | Statistic | p-value | Statistic | p-value | Statistic | p-value |
| Inverse chi-squared(378), P | 618.3729 | 0.0000 | 4051.397 | 0.0000 | 516.9447 | 0.0000 | 112.1546 | 1.000 |
| Inverse normal, Z | -1.0822 | 0.1396 | -26.5022 | 0.0000 | -2.3338 | 0.0098 | 20.4025 | 1.000 |
| Inverse logit t(949), L* | -2.5235 | 0.0059 | -70.1773 | 0.0000 | -3.1077 | 0.0010 | 21.3488 | 1.000 |
| Modified inv. chi-squared, Pm | 8.7423 | 0.0000 | 128.315 | 0.0000 | 5.0534 | 0.0000 | -10.7522 | 1.000 |
